# Secondary implant stability outcome of immediate versus late placed variable-thread implants in the maxilla. A retrospective cohort study

**DOI:** 10.4317/jced.54147

**Published:** 2017-09-01

**Authors:** Nicolas Grognard, Gino Verleye, Dimitrios Mavreas, Bart Vande-Vannet

**Affiliations:** 1 Kliniek Royal, Oostende, Belgium; PhD student @ CHIR- Unit Dentistry – ORHE, department of Orthodontics, Faculty of Medicine and Pharmacy, Vrije Universiteit Brussel, Belgium; 2 Professor, Communication Sciences, Universiteit Gent, Belgium; 3 Professor, CHIR- Unit Dentistry – ORHE, department of Orthodontics, Faculty of Medicine and Pharmacy, Vrije Universiteit Brussel, Belgium; 4 CHIR- Unit Dentistry – ORHE, department of Orthodontics, Faculty of Medicine and Pharmacy, Vrije Universiteit Brussel, Belgium

## Abstract

**Background:**

The healing of xenograft augmentated intra-alveolar gaps following immediate implant placement (IMIP) after tooth extraction is likely to differ in time and density compared to the native bone part that directly contacts the implant.

**Material and Methods:**

Secondary implant stability (SIS) data recorded 2-3 months following a late implant placement protocol (LIP) (n= 43) and 6-8 months following an immediate implant placement protocol (IMIP) (n=33) of variable-thread implants (Nobel Active™) in the maxilla were retrospectively collected from files of 63 patients (42 females, 21 males). Statistical analysis was performed using a generalized estimating equation model (GEE). Data split-up according to implant diameter (RP, Ø= 4.3mm) , narrow platform (NP, Ø= 3.5mm) was adopted.

**Results:**

For NP implants, the mean ISQ (±SD) values were 70.84 (±4.86) in LIP group and 72.41 (±3.89) in the IMIP group. For RP implants, mean ISQ (±SD) values were 73.45 (±8.77) in the LIP group and 75.93 (±5.73) in the IMIP group. Significant effect of treatment modus in favour of the IMIP and gender in favour of males and implant position was noted (*p*<0.05).

**Conclusions:**

SIS following a IMIP protocol after 6-8 months is comparable to LIP protocol after 2-3 months. A minor ISQ outcome difference in favour of the IMIP protocol can be attributed to a difference in hard tissue alteration during healing of the xenograft part.

** Key words:**Secondary implant stability, RFA, Osstell Mentor, variable thread implants, Nobel Active, Bio-Oss, immediate implant placement, late implant placement, non-submerged healing, gap.

## Introduction

The time-framing classification of implant placement after tooth extraction as proposed by Hämmerle *et al.* (1,2), defines an immediate implacement protocol (IMIP) after extraction as a type 1 procedure, whereas a late implant placement protocol (LIP) after at least 16 weeks following extraction as a type 4 procedure. In case of single hopeless teeth, especially in the esthetic zone, objective benefits to the patients can be attributed to an immediate implant placement after extraction (IMIP) protocol. These comprise mostly a one-time intervention and the possibility to offer an instant fixed provisionalization. On the other hand, pre-operative risk assessment if of utmost importance as outlined by Cosyn *et al.* (3,4). In their reports, based on 1 and 3 years results, warnings concerning adverse soft tissue factors/outcomes and recommendations to anticipate esthetic failures are clearly formulated.

One of the particular aspects following the insertion of an implant in a fresh extraction alveolus, is the presence of a ‘peri-implant’ gap situated between a part of the inserted implant outer surface, mostly buccally, and the inner lining of the fresh extraction alveolus. The hard alterations in these gaps following healing without any form of augmentation has been studied clinically and histologically (5). In this controlled study, bone healing around implants following an IMIP protocol – with an initial gap size of ≤ 2mm - versus a LIP protocol was histologically comparable after a healing period of 12 months, meaning that solely a blot clot can initiate bone healing and gap closure. Later on, the pattern of gap resolving was decribed as a process of new bone formation in the peri-implant gap area and bone resorption of the buccal and palatal bone plates in mainly horizontal direction (6). Although necessitating a flap approach, augmentation of the peri-implant gap by application of a deproteinized bone mineral of bovine origin (Bio-Oss, Geistlich) with subsequent membrane coverage will substantially reduce the amount of both horizontal and vertical bone loss compared to absence of concommitant augmentation (7). This treatment modality was further investigated by Chen *et al.* (8) , using tissue level Straumann implants, comparing gap augmention outcomes using either a bone graft, a graft plus membrane or no additional treatment, with attention to the risk of post-operative developped mucosal in relation to the bucco-lingual final position of the implant, based on surgical re-entry evalutions 6 months after implant placement. It was concluded that the risk for mucosal recession development was significantly higher with buccally positioned implants compared to lingually positioned implant. Furthermore, after a 6 months period re-entry procedure, 3 types of healing in the gap area were noted, irrespective of the type of additional treatment. Complete gap resolution, residual ‘moat’ type defects of bone dehiscence type defects were found. In general, creating intentionallty larger gaps allowing for insertion of larger volumes of Bio-Oss, anticipates horizontally directed resorption of the buccal bone plate with associated preservation of the ridge contour. A minimal flap elevation approach, alternative to the above described ones, was decribed by Derouck *et al.* in patients exhibiting a normal or thick gingival biotype (9). In their approach, buccally located marginal gaps after immediate implant placement were augmented with Bio-oss insertion between the residual socket wall and the implant without adjunctive placement of a membrane. Instant provisionalization or delayed restoration was done according to randomization procedure. They concluded that soft tissue outcome was substantially improved after instant provisionalization in terms of preservation of papilla and mid-facial mucosa compared to delayed restoration. Whether a difference exists between the healing and maturation of the bone graft of the marginal gaps between the implant and the inner wall of the alveolus with or without an adjunctive membrane in an non-submerged approach is not known. From an experimental study in dogs (10), it is known that bone healing of Bio-Oss filled gaps around implants between 3 and 7 months is characterized by mean volume of hard tissue is occupied by Bio-Oss particles with a slow resorption of the latter. Furthermore, histologic analysis of biopts harvested from Bio-Oss and membrane augmented extraction sockets reveal various stages of bone maturation and formation after 4 months. This can lead to an interpretation that bone maturation in an Bio-Oss grafted environment is probably unfinished 4-7 months after insertion (11). Therefore, it can be assumed that bone healing after immediate implant placement after with subsequent Bio-Oss augmentation of the gap, is likely to follow a dual osseointegration process. The part of the implant that is in contact with native bone will follow a normal osseointegration traject during 2-3 months, as described for moderately rough surfaced implants (12). The part of the implant that is in contact with Bio-Oss, is given the lack of systematic evidence regarding the maturation time of Bio-Oss in the present situation, unknown.

Implant stability assessment by means of resonance frequency analysis (RFA) is based on quantitative assessment of microdeflection of a tested implant in the surrounding jawbone, by aid of a transducer, induced by electromagnetic excitation. A comprehensive review of this methodology was reported by Sennerby and Meredith. The properties of the transducer, the stiffness of the implant, the stiffness of the bone and the properties and stiffness of the implant-bone complex are main RFA influencing factors. Furthermore, RFA devices possess the power to detect implant stability at the level that is not achieveable with traditional radiographical and/or clinical methods (13-15). As the above mentioned recommendations regarding implant positioning results in a rather voluminous gap, a quite extentive part of the implant is expected to be in contact with the added bone substitute after gap aumentation, directly influencing both the bone stiffness the implant-bone stiffness. The healing process in the gap zone is expected to result in bone maturation with incorporation of the bone substitute particles that with time will reinforce the implant-bone interface.

Therefore, the aim of this study was to compare the RFA based secondary implant stability outcome of maxillary placed immediate implants after extraction, subsequent Bio-Oss gap augmentation and instant provisionalization to those of identical implants placed in healed sites with delayed restoration. The null hypothesis no difference in RFA based secondary implant stability between two treatment modalities was adaopted (*p*<0.05).

## Material and Methods

-Patient selection and general treatment outline

This retrospective cohort study was conducted in a private periodontal practice. Data were retrieved from files of formerly treated patients that received unsplinted variable thread implants in the maxilla. The included patients were referred for either implant treatment in healed sites according to a late implant placement (LIP) or for implant insertion immediately after extraction in case of teeth with a negative prognosis due to fracture, caries, root resorption or endodontic complications (IMIP). In both groups, treatment planning was in part based on cbct-scan imaging and virtual planning using planning software (Nobel Clinician®, Nobel Biocare, Gothenburg, Sweden) as a preparation for free- handed implant insertion. General exclusion criteria for implant surgery including including smoking more than 10 sigarettes per day were applied for both groups. In both treatment groups, per os antibiotic profylaxis was performed using a three day administration of azithromycin 500mg (Zitromax, Pfizer, NY, NY, USA), starting 1 day before implant surgery. Post-operative home-care was performed using topical application of hyaluronic acid spray tid (Gengigel, Dental Impex Pharma BV, Leerdam, The Netherlands) and a 0.2% chlorhexidine mouthwash tid (Perioaid, Dentaid, Houten, The Netherlands) during 14 days.

The following search criteria were adopted for case selection in files of formerly treated patients.

• Periodontally healthy subjects.

• Maxillary sites.

• Referrral for either a LIP or IMIP procedure.

• No previous or concommitant bone augmentation procedures in case of the LIP group subjects.

• Uneventfull healing in the period between insertion and time of measurement.

• Implant system specifications: Nobel Active RP platform implants with a diameter of 4.3mm or NP platform implants with a diameter of 3.5mm (implant length: 10mm-15mm).

The search selection resulted in a a cohort of 63 formerly treated patients (52 females, 24 males) (mean age 56.2± 12.3 years; range: 34-78 years). These patients received in total 76 implants (LIP group: 42 implants, IMIP group: 34 implants), meaning that multiple patients received multiple implants (up to 3).

-Late implant placement protocol (LIP)

In the LIP group, a flap approach with non-submerged healing modus by aid of a healing abutment was applied by following the specific surgical protocol for Nobel Active advocated by the manufacturer. Hand-driven insertion using the specific designed Nobel Active surgical handle was used systematically.

-Immediate implant placement protocol (IMIP)

In the IMIP group, in general, a flapless approach after tooth extraction for immediate implant placement was used. The residual gap was filled in combination of intra-alveolar augmentation with a particulated, deproteinized, bovine derived bone substitute material (Bio-Oss®, Geistlich Pharma AG, Wolhusen, Swiss) was applied in the residual gap and a non-functionally loaded provisionalisation by aid of a temporary abutment (Quick Temp, Nobel Biocare, Gothenburg, Sweden) was provided. In detail, a-traumatic and gentle tooth extraction was performed using appropriate elevators, luxators and eventually an ultrasonic surgery device (Satelec Piezotome 2, Acteon Group, Merignac, France). After tooth extraction, carefull inspection and debridement of the alveolus was performed under constant irrigation with a physiologic saline solution. Correct three-dimensional implant positioning was performed according to guidelines of Buser *et al.* (16) and Chen *et al.* (8). Acording to the latter, implant positioning resulting in a minimal horizontal gap distance of 2mm was anticipated using the specific recommended hand-driven insertion protocol for the given implant system by aid of a long implant driver handle. Subsequent application of the particulated bone substitute in to the gap was done using specially designed curve-shaped bone compactors (Denteo, BioTech Dental, St. Quentin-Fallavier, France). During this, the internal implant part was sealed using a cover screw. Intentional ‘overfill’ of the of the gap was performed according to the guidelines of Graauwmans *et al.* (17). After gap obturation, the cover screw was removed and a Quick Temp abutment with appropriate height (1.5mm or 3mm) was choosen depending on the final vertical implant position in relation to the distance of the soft tissue margins. The Quick Temp was hand torqued. A provisional crown was prepared by either using the natural crown or a present PFM crown after root separation and subsequent relining with incorporation of the PEEK cap by aid of a resin-glasionomer dual curing material (Geristore, Denmat, Lompac, CA, USA ) or using a customized transparent strip crown (Frasaco Gmbh, Tettnang, Germany). In case of use of the natural crown or PFM, a putty material impression (Optosil, Hereaus-Kulzer BV, Haarlem, The Netherlands) was prepared before tooth extraction and used to reposition the crown in its proper position during relining. After proper emergence profile preparation and polishing, the temporary crown was cemented on the Quick Temp abutment using a temporary cement (Temp Bond, Kerr Dental, Orange, CA, USA). A regular intrabuccal radiograph was taken after cement setting to detect cement remnants. After meticuluous remnant removal, non-resorbable intra-papillary sutures were placed (Dafilon 4/0-5/0, B. Braun Medical NV, Diegem, Belgium). Occlusion and articulation was reduced to complete non-contact.

-Stability measurements

The collected secondary implant stability data were performed after a mean healing period of approximately 2-3 months in LIP group and after approximately 6-8 months in the IMIP group. The latter was assumed to be justified by the presumed maturation of the Bio-Oss xenograft of approximately 6 to 8 months for procedural simplicity. RFA measurements were performed on each implant at the implant level using a wireless type Osstell Mentor device (Osstell AB, Gothenburg, Sweden). An implant-specific transducer, called ‘Smartpeg’ (Osstell AB, Gothenburg, Sweden) was used for each implant type. For NP implants, Smartpeg type 61 was used. For RP implants, Smartpeg type 60 was used. RFA data are reported in implant stability quotient units (isq), wherein greater positive values indicate greater stability. Measurements were repeated until a constant value was obtained. The last (consistently obtained) value was used for statistical analysis. No data concerning primary stability or bone quality were present for analysis.

-Statistical analysis

The SPSS statistical software package 22.0 (IBM SPSS, Chicago, USA) was used. Since multiple patients received up to three implants, a possible unknown correlation between secondary implant stability outcomes was anticipated by application of ‘Generalized Estimation Equation’ statistical modelling. Furthermore, data split-up into 2 subgroups according to implant platform (NP: 3.5mm Ø and RP: 4.3mm Ø) was done. Mean estimated secondary implant stability values were presented according to the GEE modelling with standard errors and 95% confidence intervals (CI). The effects of gender, implant type and implant position (incisor, canine or premolar regions) as covariates on the implant stability outcome was investigated after GEE modelling. The null hypothesis of no effect in outcome for various both patient and implant related dependent variables was adopted. The level of significance was set at *p* = 0.05.

## Results

A cohort of 63 formerly treated patients (52 females, 24 males) (mean age 56.2± 12.3 years; range: 34-78 years) was withheld for analysis. These patients received in total 76 implants (LIP group: 42 implants, IMIP group: 34 implants), meaning that multiple patients received multiple implants (1-3).

The distribution of cases in the IMIP group according to reason for tooth extraction and insertion location are depicted in  Table 1. Toothfracture was the major reason of tooth loss (76%). The maxillary incisor and premolar regions received most of the immediate placed implants.

**Table 1:**
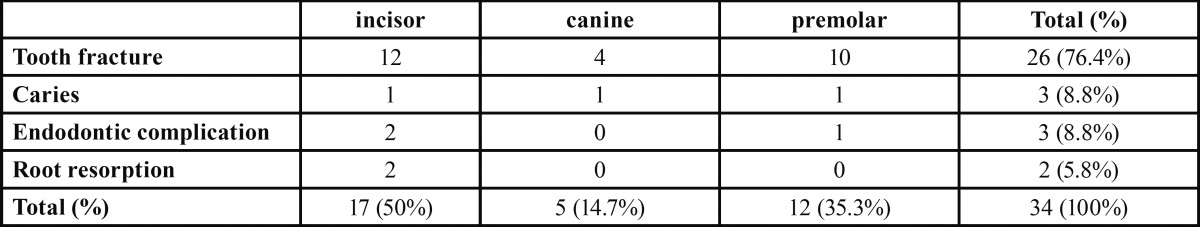
Distributions of maxillary positions of immediately placed implants and reasons for extractions.

The estimated mean (±SD) values of RFA based secondary implant stability for both treatment modalities according to implant diameter are depicted in  Table 2. GEE based analysis revealed no statistical difference between both treatment modalities for each given implant diameter. The effect of implant and patient related coviarates on the secondary implant stability is shown in  Table 3. GEE modelling revealed no effect of implant diameter, implant length or healing time on the ISQ outcome . The effect of implant position on the outcome was statistically significant in favor of posterior sites compared to anterior site with a mean difference of 4 ISQ (*p*= 0.045). The effect of gender was highly significant in favor of males compared to females with a mean difference of 5 ISQ (*p*=0.000).

**Table 2:**
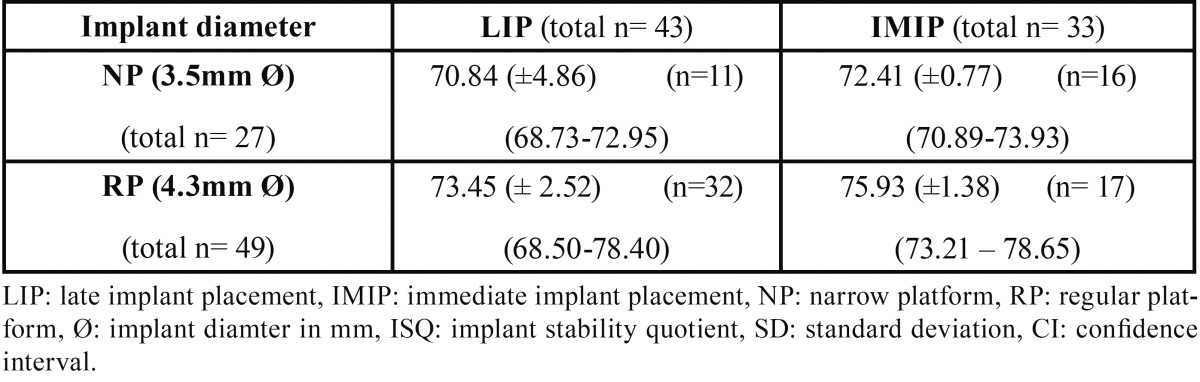
Mean estimatess (ISQ units) (+- SD) and 95% CI’s of RFA based secondary implant stability according to implant diameter and procedure.

**Table 3:**
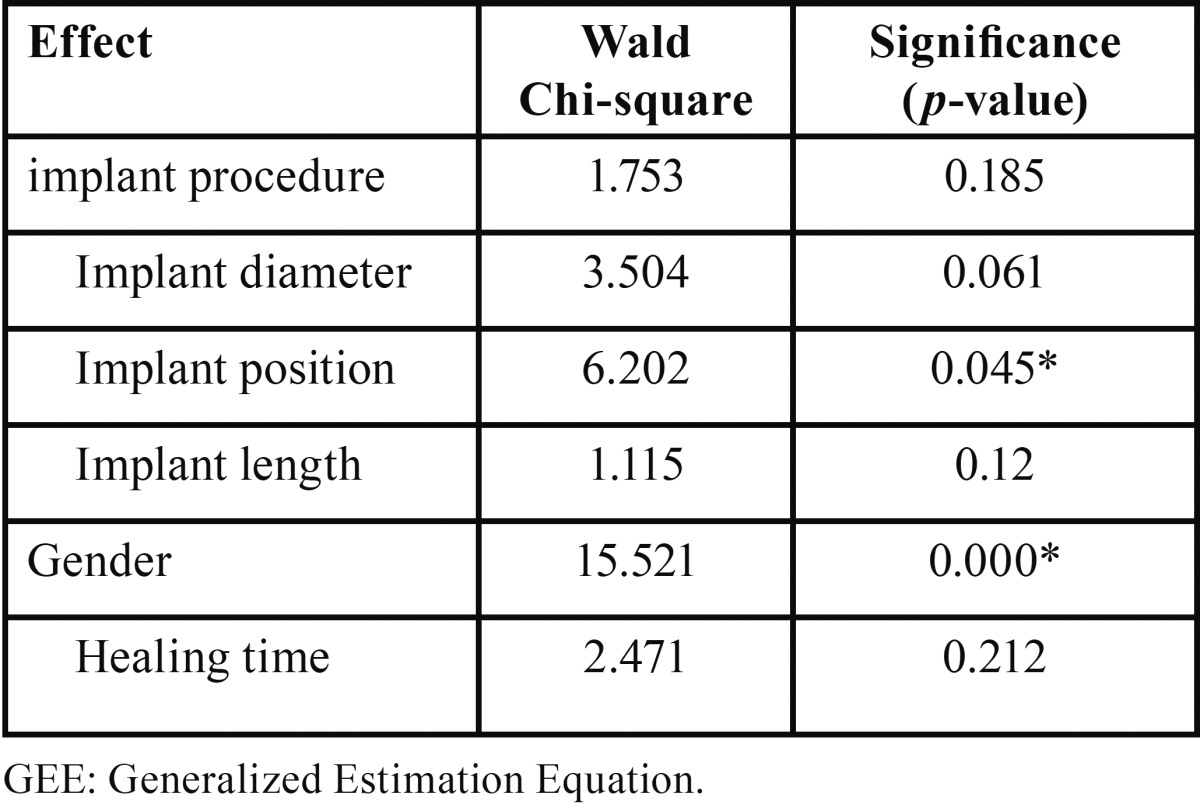
Effects of procedure, implant diameter, implant position, length and healing timime on stability measurements according to the GEE modelling.

## Discussion

The assessment of treatment outcome of implants, placed immediate after tooth extraction in a fresh alveolus in the esthetic zone, is a task with high input of esthetic driven evaluation factors. Among these, short and long term outcome of soft tissue aspects concerning papillary and mid-facial mucosal dimensions is obvious (3,4,8). Since soft and hard tissue healing aspects are in part influenced by correct palatally oriented implant positioning, the presence of a quite extensive post-surgical marginal gap is likely to be present. This implies that a reduced part of the implant circumference will contact native bone. In order to achieve sufficient primary stability, adequate preparation and insertion technique are important, together with the choice of an implant design that offers suiteable primary stability. In a recent retrospective study, the primary stability of various implant systems was compared (18). Tapered Nobel implants, both progressive and uniformely treated, showed highest rotational stability characteristics compared to implant systems of other manufacturers.

The effect of the presence of a condensed, particulated bone substitute in the gap on the primary stability is at present unknown. Moreover, as outlined in the introduction, the evolution and timing of bone maturation in the augmented gap area without subsequent placement of a membrane is unclear. Determination of implant stability by means of electronic devices that are able to detect and monitor subclinical changes during the osseointegration period can be helpfull beside traditional clinical and radiographical examination. In referred settings, knowledge of secondary implant stability values obtained after the presumed period of active osseointegration is more important for the restorative clinician than information regarding primary stability. For several contempory moderately rough implant surfaces, a period of 2-3 months is decribed to be accurate to accomplish normal osseointegration during an uneventfull healing process (12), meaning that implants placed following a late implant placement protocol in healed sites (LIP) can be screened for secondary implant stability. In case of implants placed immediately after extraction, as described a dual healing process is to be expected, since the bone maturation along the inserted bone substitute particles is presumed to follow a different and more time consuming pattern. As described in the introduction, given the lack of systematic evidence regarding the time-framing of bone healing of membrane uncovered Bio-Oss bone substitutes, an arbitrary choosen period of 6-8 months was choosen as the post-insertion period to assess secondary implant stability for implants following IMIP protocol. The choice of a 6-8 months healing period seems justified based on the finding of Chen *et al.*

In this study, based on retrospectively collected data of patients referred for implant therapy for either LIP or IMIP in the maxilla, secondary implant stability assessed by a Osstelll Mentor were compared. In other to produce a clear data set, data of only one specific implant system, applied in both groups, were analyzed. Furthermore, data split with respect to implant diameter was done to exclude confounding by this particular factor. For both diameters, comparable RFA based stability outcomes were noted between both groups, slightly in favor of the implants following the immediate implant protocol. As expected the ISQ values were for both treatment modalities slightly lower after use of 3.5mm Ø implants compared to 4.3mm Ø implants. In an attempt to compare the noted outcomes, only a report concerning RFA based implant stability values of RP 4.3mm Ø Nobel Active applied in posterior sites of the mandible were found (19). In this study, baseline values of 78.49 (±2.35) ISQ and 4 month post-insertion values of 81.50 (±1.91) ISQ were noted. Compared to the 4.3 mm Ø ISQ outcomes in the present study, a difference magnitude of 5-8 ISQ units is present. This in well line with reported outcome differences between upper and lower inserted of another implant system (Straumann SLA tissue level 4.1mm RN implants) (20,21). Whether the noted small, clinically non relevant, ISQ differences in the present study between both treatment modalities in favor of the implants following the IMIP protocol compared to the LIP protocol can be attributed to a difference of bone density in area of the Bio-Oss material augmented gap area is at present unclear. Although not published in a peer-reviewed English journal, the publication of Graauwmans *et al.* (17) deserves attention. In this study, cbct-scan evaluations were done after immediate implants placement in the anterior part of the maxilla with focus on changes of the mid-facial aspect of the buccal bone plate starting from extraction until 4 years post insertion. In this study Bio-Oss was used as the solely bone substitute augmentation without concomitant use of a membrane in cases of a 2mm installed horizontal gap size. The immediate post-operative bone plate thickness increased from 0.9mm to 2.4mm due to the presence of the Bio-Oss bone substitute. Although during the evaluation period between 1 and 4 years, a decrease of the mid-facial buccal bone plate was noted by 1.8mm, surprisingly, an increase of the buccal plate height of approximately 1.2mm was noted.

In summary, in the present study, short term secondary implant stability data are presented and compared for Nobel Active implants placed following type 1 and type 4 post extraction protocols. The results point to comparable results in secondary implant stability outcomes between both treatment modalities. Small subclinical differences in ISQ outcome were detected between both treatment modalities by the Osstelll Mentor. In a long term perspective, other criteria to assess the overall outcome of a type 1 treatment modality are obvious. In an attempt to systematically review the available literature concerning the clinical outcomes and incidence of complications associated with immediate and early placed implants (type 2), Quirynen *et al.* (22) reported a <5% implant loss for both treatment modalities but although a wider range for type 1 implants (0% - 40 %) compared to type 2 implants (0% - 9%).

Furthermore, the latter authors stated that due to heterenogenity of information in the analyzed papers, uncertainties remain regarding peri-implant health, bone stability and esthetic outcome concerning type 1 implants. Bell *et al.* (23) reported success rate for Nobel Active implants used in type 1 and type 4 post extraction protocols. A success rate difference of approximately 5.6% was found between both treatment modalities in favor of type 4 protocols irrespective of comparable primary stability in terms of insertion torque. These conclusions were further amplified by Cosyn *et al.* (24) based on 5-year prospective findings of single immediate implants placed in the esthetic zone. In a cohort of 17 well selected patients, treated by experienced clinicians, an esthetic complication of 47% was noted in terms of mid-facial recession, mid-facial contour and alveolar process deficiency.

## Conclusions

Within the limits of this study (sample size and lack of data regarding availability of patterns of evolution of implant stability during the osseointegration period), it appears that the RFA based secondary implant outcome of immediate placed single, unsplinted, variable-thread implants in the maxilla with subsequent gap augmentation and instant non-occlusal provisionalization, is comparable with those of similar implants placed according to a late implant placement protocol without instant provisionalization. Moreover, a slightly higher clinically non-relevant RFA outcome was noted in favor of the immediately placed implants. This difference is possibly explained by a higher density of the healed bone substitute applied in the immediate implant protocol at the buccal gap zone. The reported RFA based secondary implant stability values for the investigated implant system and the given treatment protocol can serve as indicative values for future research and comparison.
